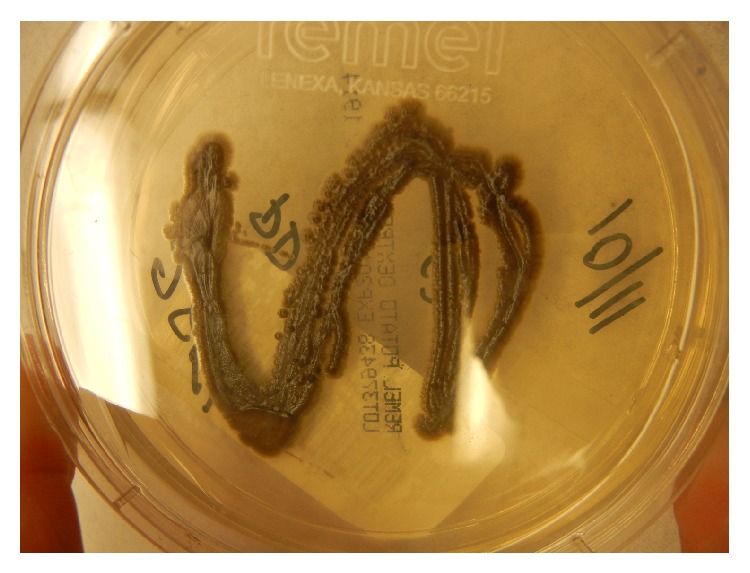# *Exophiala* Pneumonia Presenting with a Cough Productive of Black Sputum

**DOI:** 10.1155/2015/821049

**Published:** 2015-05-05

**Authors:** Yehuda Z. Cohen, Wendy Stead

**Affiliations:** Division of Infectious Diseases, Department of Medicine, Beth Israel Deaconess Medical Center, Harvard Medical School, Boston, MA 02215, USA

## Abstract

*Exophiala* species are black, yeast-like molds that can cause subcutaneous cysts as well as disseminated disease. Isolated pneumonia due to *Exophiala* species is extremely uncommon. We report a case of isolated *Exophiala* pneumonia in a patient with bronchiectasis who presented with worsening dyspnea and a cough productive of black sputum. The production of black sputum, known as melanoptysis, is an uncommon physical finding with a limited differential diagnosis. To our knowledge, this is the first reported case of *Exophiala* pneumonia presenting with a cough productive of black sputum.

## 1. Introduction

*Exophiala* species are dematiaceous (dark-pigmented) environmental fungi with a worldwide distribution. They are often described as black, yeast-like molds [1]. Infection with* Exophiala* species is uncommon but typically presents as subcutaneous cysts and can occur in both immunocompetent and immunocompromised individuals [2]. Disseminated disease is often neurotropic and carries a poor prognosis [3, 4]. Isolated pneumonia due to* Exophiala* species is extremely rare. Although* Exophiala dermatitidis* has been found to colonize the airways of up to 19% of patients with cystic fibrosis [5, 6], only a few cases of* Exophiala dermatitidis* pneumonia have been reported in that population [7–9]. A small number of cases of* Exophiala dermatitidis* pneumonia, as well as a single case of* Exophiala jeanselmei* pneumonia, have also been reported in patients without cystic fibrosis [10–13]. Here we present a case of* Exophiala* pneumonia in a patient with bronchiectasis who presented with a cough productive of black sputum.

## 2. Case Presentation

**Figure 1:**
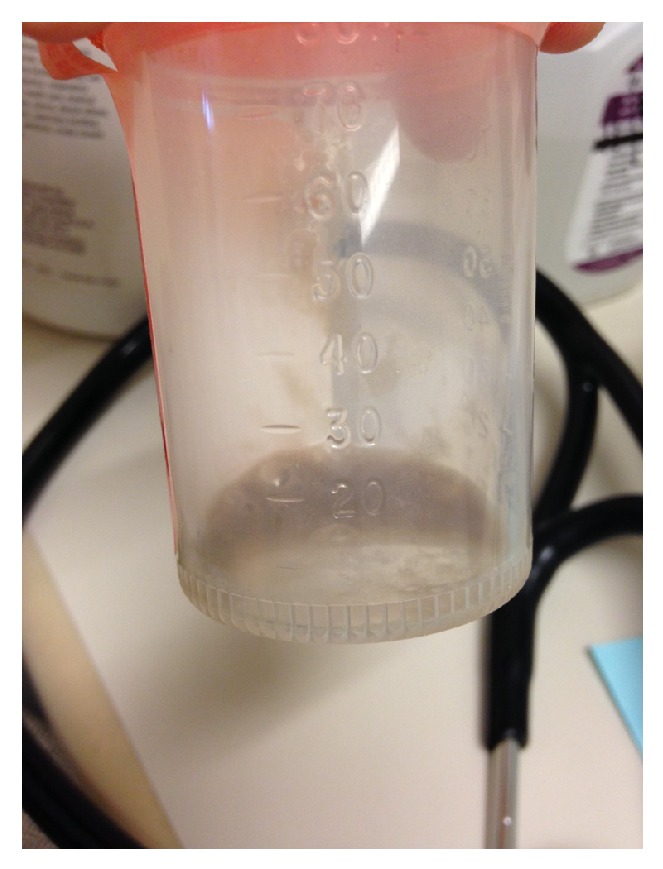


A 75-year-old woman with bronchiectasis was referred to the infectious diseases clinic with worsening dyspnea on exertion and a productive cough over the previous 3 months. She had been diagnosed with bronchiectasis about 30 years earlier and was doing well until 7 years prior to presentation when she was diagnosed with pulmonary* Mycobacterium avium* complex infection. She was treated with clarithromycin, rifampin, and ethambutol for over 2 years with improvement in symptoms. Over the next few years, dyspnea on exertion and mucus production slowly increased. During the 5 years prior to presentation, 12 respiratory cultures for acid-fast bacilli were performed, and all were negative. Seven fungal respiratory cultures were performed during this time as well, and all returned positive for* Exophiala jeanselmei*, which was thought to represent colonization. These isolates were identified based on morphology, which is known to be unreliable [14]. In the 3 months before presentation, dyspnea on exertion and cough significantly worsened, and the patient began to produce black sputum (Figure 1). Physical examination was unremarkable. A thoracic CT scan revealed bronchiectasis that was unchanged and new consolidations in the lower lobes. Sputum acid-fast culture was negative. Sputum fungal culture grew a black, yeast-like mold (Figure 2), which was again identified on the basis of morphological characteristics as* Exophiala jeanselmei*. Susceptibility testing demonstrated an MIC of 0.5 *μ*g/mL for amphotericin B, 0.5 *μ*g/mL for itraconazole, and 0.25 *μ*g/mL for voriconazole. The patient was started on 200 mg of itraconazole daily. After 6 weeks of therapy, the patient was no longer producing black sputum and began to have some improvement in her symptoms. By 5 months of therapy, the patient had experienced a dramatic improvement in symptoms. Therapy was continued for a total of 6 months.

Three weeks after stopping itraconazole the patient again developed increased shortness of breath and cough productive of black sputum. Sputum fungal culture at that time grew an isolate morphologically identified as* Exophiala dermatitidis*. DNA sequencing of the ITS and D1/D2 regions confirmed the specimen to be* Exophiala dermatitidis*. Susceptibility testing revealed an MIC of 1 *μ*g/mL for amphotericin B, 0.5 *μ*g/mL for itraconazole, 0.25 *μ*g/mL for posaconazole, <0.03 *μ*g/mL for voriconazole, and 0.015 *μ*g/mL for terbinafine. The patient was started on voriconazole 200 mg twice daily with improvement in symptoms and resolution of black sputum production, with a plan to continue therapy for at least one year. Therapy was stopped after 7 months when the patient developed alopecia and peripheral neuropathy. Posaconazole 300 mg twice daily was started but discontinued after 2 months when additional neurologic symptoms developed. The patient opted not to pursue any further antifungal treatment at that time.

## 3. Discussion

Only a handful of cases of* Exophiala* pneumonia have been reported in the literature. These include 3 cases in patients with cystic fibrosis [7–9], 2 cases in patients with bronchiectasis [10, 11], and 2 cases in patients without prior lung disease [12, 13].* Exophiala dermatitidis* is known to colonize the airways of individuals with cystic fibrosis, but no studies have been performed to investigate if* Exophiala dermatitidis* also commonly colonizes the airways of patients with noncystic fibrosis bronchiectasis. In our patient,* Exophiala* species had been present in respiratory cultures for 5 years prior to her presentation. It seems likely that the patient was colonized with this organism for many years and that infection only occurred in the few months before presentation when her symptoms considerably worsened, the production of black sputum began, and consolidations were found on CT. Treatment with antifungals resulted in improvement of symptoms and resolution of black sputum production.

In this patient,* Exophiala* isolates from sputum cultures were originally identified as* Exophiala jeanselmei* and later as* Exophiala dermatitidis*. DNA sequencing confirmed the later isolate to indeed be* Exophiala dermatitidis*. It appears unlikely that this patient had a mixed infection with 2* Exophiala* species. The more likely scenario is that* Exophiala dermatitidis* was the sole etiologic agent, and the original isolates, which were identified on the basis of morphological characteristics only, were misidentified.

Although* Exophiala* species produce melanin and appear black, no prior reports of* Exophiala* pneumonia have mentioned black sputum as a presenting feature. The production of black sputum, termed melanoptysis, is an uncommon clinical finding in general and has a limited differential diagnosis. It appears to occur most often in coal miners with progressive massive fibrosis and cavitary pneumoconiosis [15]. It has also been described in smokers of alkaloid cocaine (crack) [16] and as a result of malignant melanoma [17]. Infection with another fungus that produces melanin,* Aspergillus niger*, has also been reported as a cause of melanoptysis [18]. Interestingly, a patient who presented with* Exophiala dermatitidis* subconjunctival mycetoma was reported to have black deposits in her tears [19].

In conclusion, isolated* Exophiala* pneumonia is a rare cause of pneumonia that appears to occur more frequently in individuals with cystic fibrosis or bronchiectasis. The production of black sputum, termed melanoptysis, is an uncommon physical finding.* Exophiala* pneumonia can be added to the differential diagnosis in patients who present with this unusual finding.

## Figures and Tables

**Figure 2:**